# The influence of disease categories on gene candidate predictions from model organism phenotypes

**DOI:** 10.1186/2041-1480-5-S1-S4

**Published:** 2014-06-03

**Authors:** Anika Oellrich, Sebastian Koehler, Nicole Washington, Sanger Mouse Genetic Project, Chris Mungall, Suzanna Lewis, Melissa Haendel, Peter N Robinson, Damian Smedley

**Affiliations:** 1 Wellcome Trust Sanger Institute, Wellcome Trust Genome Campus, CB10 1SA Hinxton, UK; 2 Institute for Medical Genetics and Human Genetics, Universitaetsklinikum Charite, Augustenburger Platz 1, 13353 Berlin, Germany; 3 Berkeley Bioinformatics Open-Source Projects, Lawrence Berkeley National Laboratory, 1 Cyclotron Road, CA 94720 Berkeley, USA; 4 Ontology Development Group, OHSU Library, Oregon Health & Science University, 3181 S.W. Sam Jackson Park Rd, OR 97239 Portland, USA

## Abstract

**Background:**

The molecular etiology is still to be identified for about half of the currently described Mendelian diseases in humans, thereby hindering efforts to find treatments or preventive measures. Advances, such as new sequencing technologies, have led to increasing amounts of data becoming available with which to address the problem of identifying disease genes. Therefore, automated methods are needed that reliably predict disease gene candidates based on available data. We have recently developed Exomiser as a tool for identifying causative variants from exome analysis results by filtering and prioritising using a number of criteria including the phenotype similarity between the disease and mouse mutants involving the gene candidates. Initial investigations revealed a variation in performance for different medical categories of disease, due in part to a varying contribution of the phenotype scoring component.

**Results:**

In this study, we further analyse the performance of our cross-species phenotype matching algorithm, and examine in more detail the reasons why disease gene filtering based on phenotype data works better for certain disease categories than others. We found that in addition to misleading phenotype alignments between species, some disease categories are still more amenable to automated predictions than others, and that this often ties in with community perceptions on how well the organism *works *as model.

**Conclusions:**

In conclusion, our automated disease gene candidate predictions are highly dependent on the organism used for the predictions and the disease category being studied. Future work on computational disease gene prediction using phenotype data would benefit from methods that take into account the disease category and the source of model organism data.

## Background

Despite many success stories in the identification of genetic causes for human heritable diseases, half of the currently described disorders with a presumed genetic etiology are still without an identified molecular basis [1]. Although the identification of a novel disease gene rarely leads to immediate, novel treatment options, clearly an understanding of the cellular pathways and networks affected by a genetic mutation is the basis for developing improved treatment strategies and optimal genetic counseling. To support the identification of genetic causes, and with that treatment of human heritable disorders, biological as well as computational methods have been developed [2-7]. However, none of the existing solutions is capable of providing reliable answers for all diseases and improvements are still needed.

Technology advances have led to solutions enabling rapid and cheap identification of variants in human genomes and exomes. However, these methods yield long lists of variants reflecting the fact that each individual harbours more than 30,000 variants identifiable by exome sequencing, with typically 5% or more of variants not being listed in databases of variants such as dbSNP. Typical bioinformatic filtering procedures remove common variants and those deemed to be nonpathogenic, but are not able to narrow the search down to only a short list of candidates based only on the sequence variants.

In a recent study, we presented the PHenotypic Interpretation of Variants in Exomes (PHIVE) algorithm that in addition to traditional variant filtering and evaluation also includes the phenotype manifestations in individuals as well as the signs and symptoms of diseases [8]. It was shown that including phenotype information into the prioritisation of candidate genes leads to an up to 54.1 fold improvement over methods purely based on variant information. To assess the phenotypic suitability of a gene variant, PhenoDigm's phenotype comparison algorithm was used [4]. The study also showed that the performance of the PHIVE algorithm is influenced by the mode of inheritance (autosomal dominant vs. autosomal recessive) and by the class of mutation (nonsense and missense mutations). However, our investigations did not include an evaluation of the characteristics of the diseases in question.

Ongoing debates highlight that model organisms do not necessarily constitute ideal fits for certain diseases [9], due to e.g. changes in gene expression [10], but can still provide valuable insights into a disease even though only part of the phenotypes may be reproduced in a model organism [11,12]. To facilitate the linkage of model organisms and diseases, ongoing efforts such as the International Mouse Phenotyping Consortium (IMPC) and the Zebrafish Mutation Project (ZMP) record a range of predefined parameters that are not just restricted to phenotypes related to the disease area a researcher is studying [13].

The number of automated disease gene candidate prediction tools using cross-species information is also increasing [14,4,6,5]. The aforementioned tools rely on the availability of logical definitions for phenotypes that allow their comparison across species [15]. Typically, precision and recall measures for known gene-disease association are reported to give an indication about the potential of the method and its suitability to the task of disease gene candidate identification. While Börnigen et al. worked on unbiased evaluation of the tools [16], to our knowledge, no further evaluation for performance of different disease categories has been undertaken. Tools that use model organism data for the prediction are limited not only to the availability of sufficient and unbiased experimental data, but are also restricted to disease areas where model organisms recapitulate the disease and where the phenotype associated genes are orthologous.

In this study, we analysed Exomiser's performance with respect to disease categories provided by Orphanet [17]. As the performance of Exomiser is influenced by the PhenoDigm phenotype comparison algorithm, we based our experiments on the evaluation of PhenoDigm and its applicability to disease categories. Using known gene-disease associations in the Orphanet and Online Mendelian Inheritance in Man (OMIM) databases [1], we identified areas for further improvements that will consequently influence Exomiser's performance. Although we only currently use PhenoDigm's mouse-based predictions in Exomiser, we plan to take advantage of zebrafish phenotypes amongst other model organism data in the future as part of our participation in the Monarch Initiative. Hence, we performed our assessment across both mouse and zebrafish data.

One factor in the poorly performing disease categories was the sub-optimal implementation of our approach for certain phenotype annotations, leading to missing phenotype alignments. These will be addressed in future releases. Other clinical phenotypes were not matched because they can not be accurately observed in the model organism in question. Interestingly, some perceptions of how well or how easily different model organisms can be fitted to particular disease areas are mirrored in the evaluation results. We conclude that automated prediction methods could potentially benefit from taking into consideration the categories of disease in which semantic model organism phenotype matching works best.

## Results and discussion

**Figure 1:**
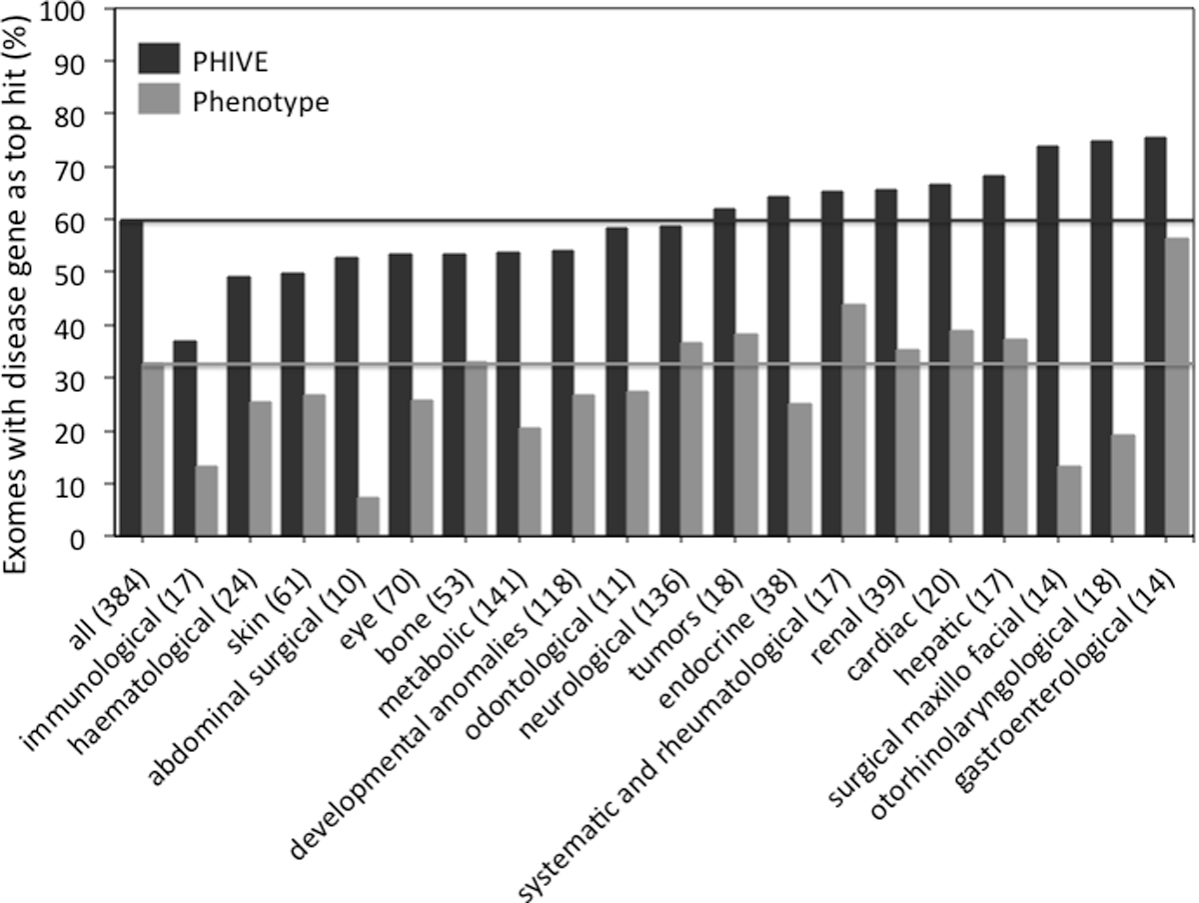
**Performance of PHIVE score in Exomiser by Orphanet disease category, together with just the phenotype-based scores**. The number of diseases tested for each category are shown in parentheses. Note that many diseases belong to multiple disease categories. The overall PHIVE performance and the contribution of the phenotype score used in Exomiser is seen to vary with respect to the disease category. The highest contribution of the phenotype score is in the category of *gastroenterological *diseases, the smallest in the category of *abdominal surgical *diseases.

Exomiser provides functionality to filter and prioritize gene variant lists using our PHIVE algorithm which combines phenotype comparisons from PhenoDigm in addition to allele frequency and pathogenicity scores [8]. Our benchmarking of Exomiser was based on 28,516 known disease-causing mutations from the Human Gene Mutation Database [18]. Using Orphanet's disease categorisation [19], we further divided Exomiser's evaluation exome data sets by disease category. Figure 1 shows that Exomiser's ability to identify the disease causing genes using the PHIVE algorithm varies for the different disease categories. All results fall into the range of 35 to 78%, with best performance in the *gastroenterological diseases *category. Figure 1 also shows the performance of Exomiser if only the phenotype prioritization for genes is used but not allele frequency and pathogenicity. It is apparent that the phenotype score works better for some disease categories than for others. For example, in the case of *gastroenterological diseases *the phenotype comparison seems to contribute a lot to the identification of disease gene candidates while in the case of *surgical maxillo facial diseases*, the contribution seems to be comparatively small. Note that not all of Orphanet's disease categories are represented due to the limited coverage in our evaluation set of 28,516 known disease-causing mutations.

As the PHIVE algorithm combines PhenoDigm, allele frequency and pathogenicity prioritisation as well as some pre-filtering steps, evaluation of just the performance of the phenotype comparison is problematic. Therefore, we decided to further investigate the effect of disease category on phenotype comparisons by just looking at the performance of PhenoDigm. Due to the inclusion of these other steps in Exomiser, we do not expect observations based on PhenoDigm performance for different disease categories to translate directly to Exomiser but should indicate potential categories where we may see enhanced or reduced performance. In addition, PhenoDigm covers all of OMIM and Orphanet as well as including zebrafish so we were able to perform a more extensive evaluation of the influence of disease categories on gene candidate predictions from model organism phenotypes.

Analogous to the evaluation of Exomiser, we divided the diseases covered in PhenoDigm into the disease categories provided by Orphanet and known gene-disease associations contained in Orphanet and OMIM. We then assessed precision and recall over the different ranks of diseases genes and determined the Area Under Curve (AUC) of the corresponding Receiver Operating Characteristic (ROC) curve. The AUC measures obtained for the individual disease categories and both the species (mouse and zebrafish) are presented in Table 1. AUC measures in mouse vary in the range of [0.774, 0.901] and in zebrafish in the range of [0.540, 0.835]. These results show that AUC measures calculated over all diseases may mask disease categories that are performing well, e.g. zebrafish performs better than mouse for *cardiac malformations *although the overall performance is much worse.

**Table 1:** PhenoDigm performs best for urogenital diseases for mouse and cardiac malformation for fish out of 31 disease categories.

disease category*	diseases(mouse)†	AUC(mouse)‡	diseases(fish)†	AUC(fish)‡
abdominal surgical	104	0.856 (0.336)	67	**0.716 (0.033)**
Allergic	5	-	0	-
Bone	368	**0.870 (0.002)**	185	0.650 (0.110)
cardiac	128	0.857 (0.138)	58	**0.675 (0.049)**
cardiac malformations	34	0.822 (0.221)	23	**0.835 (1E-4)**
circulatory system	63	0.825 (0.239)	31	0.658 (0.417)
developmental anomalies in embryogenesis	943	0.852 (0.177)	475	**0.673 (1E-4)**
endocrine	307	**0.874 (0.029)**	128	0.629 (0.382)
eye	582	**0.864 (0.034)**	269	0.646 (0.147)
gastroenterological	74	0.842 (0.391)	36	**0.739 (0.031)**
haematological	151	0.816 (0.151)	53	0.603 (0.215)
hepatic	41	**0.774 (0.011)**	8	-
immunological	134	0.843 (0.391)	36	**0.540 (0.014)**
inborn errors of metabolism	384	**0.789 (5E-9)**	91	0.646 (0.103)
infectious	3	-	2	-
infertility	41	0.817 (0.154)	18	0.635 (0.496)
neurological	777	**0.787 (2E-11)**	328	0.630 (0.486)
odontological	44	0.899 (0.078)	18	0.693 (0.161)
otorhinolaryngological	150	**0.890 (0.043)**	74	**0.731 (0.015)**
renal	277	0.846 (0.479)	130	**0.676 (0.048)**
respiratory	65	0.808 (0.126)	35	0.594 (0.135)
skin	418	0.852 (0.161)	154	0.636 (0.442)
surgical maxillo facial	89	0.836 (0.367)	56	**0.723 (5E-4)**
surgical thoracic	33	0.816 (0.176)	12	0.641 (0.364)
systematic and rheumatological	68	0.832 (0.297)	14	0.592 (0.311)
teratologic	1	-	1	-
tumors	239	0.835 (0.388)	130	**0.677 (0.044)**
urogenital	62	**0.901 (0.050)**	31	0.608 (0.207)

all diseases	3728	0.845	1558	0.630

* disease categories according to Orphanet [19]; † number of diseases falling into this category with phenotype data for the orthologue(s) of the associated gene; ‡ AUC, measured on disease-gene associations from OMIM's MorbidMap and Orphanet curation. Value in brackets shows the p-value of obtained this result compared to those obtained from randomly selecting the same number of diseases. Significant results (p value *<*0.05) are shown in bold. Note that one disease may fall into different categories due to multiple systems affected by disease.

Comparing the two model organisms, the most striking observations are first of all, that the performance for zebrafish for nearly all disease categories is reduced and secondly, that performance is much more dependent on the disease category than it is for the mouse. Given the species-divide between human and zebrafish compared to mouse, some of this reduced performance and increased variability maybe expected. Although many of the organ systems and biological processes are similar in the zebrafish, some differences obviously exist that will affect certain disease categories more than others. However, much of the difference could also be due to focus of research using these different model organisms as well as varying technical difficulties with applying our semantic comparison approach to the different phenotype ontologies used for human, mouse and fish.

An additional factor, may be the extra difficulty of assigning orthology between human and zebrafish genes due to the greater evolutionary distance. In addition, many of the genes are part of a genome duplication event in zebrafish so that one human disease gene may correspond to two zebrafish genes and it may take disruption of both to recapitulate the clinical phenotypes.

To investigate some of these issues we analysed the annotations and the corresponding PhenoDigm matches in more detail for the best, intermediate and worst performing disease categories. In addition to the calculation of AUC measures, we further investigated six of the disease categories (three for each species) to obtain a better understanding of the shortcomings of either the method or the data. For each disease category, we investigated the 10 most common clinical phenotypes and their best matches in the model organism phenotypes.

### Investigation of mouse model prediction results

To identify reasons for the differences in performances with respect to the applied disease categories, we further investigated the following three categories of diseases: *urogenital, hepatic *and *neurological *diseases. We studied their annotations and the corresponding best phenotype matches in mouse produced by PhenoDigm, together with potential biological reasons for differences in performance. The results for each of the three further investigated disease categories are shown in Additional File 1 and discussed in more detail in the following sections.

#### Urogenital diseases

The ten most frequently occurring clinical Human Phenotype Ontology (HPO) phenotypes in this category include expected urogenital phenotypes such as *Cryptorchidism *as well as others such as *Short stature, Microcephaly *and *Cognitive impairment*. The latter are due to the Orphanet classification allowing diseases to be assigned to multiple categories e.g. many diseases may be classified as both urogenital and neurological leading to a preponderance of both urogenital and neurological phenotypes when looking at each individual category.

The common urogenital and other types of clinical phenotypes all matched the expected mouse phenotypes in Mammalian Phenotype Ontology (MP) (and their more specific child terms when present) with the exception of *Cognitive impairment. Cognitive impairment *was the 10th most commonly observed clinical phenotype in this disease category and is obviously a more difficult phenotype to measure in a mouse model than physical abnormalities such as *Cryptorchidism *and there is no directly corresponding MP equivalent term. Hence, PhenoDigm ends up matching numerous general abnormalities of higher mental function such as *increased anxietyrelated response *(MP:0001363) which will lead to *Cognitive impairment *not being an informative phenotype for selecting specific mouse model matches.

The specific recall for most of the associated clinical phenotypes increases the likelihood of mouse models being predicted that are relevant to the *urogenital diseases*. Assuming also, that mouse models disrupting the known urogenital disease genes produce a pheno-copy of the disease, then performance of PhenoDigm would be expected to be good for this category as was observed.

#### Hepatic diseases

Useful animal models of liver disease have only very recently been identified [20], so it is perhaps not surprising that we found that this disease category to be the worst performing for mouse. Despite the fact that mice do not necessarily constitute a "good" model organism to study hepatic diseases, we still investigated the phenotype matches together with the predicted disease models for this disease category.

**Figure 2:**
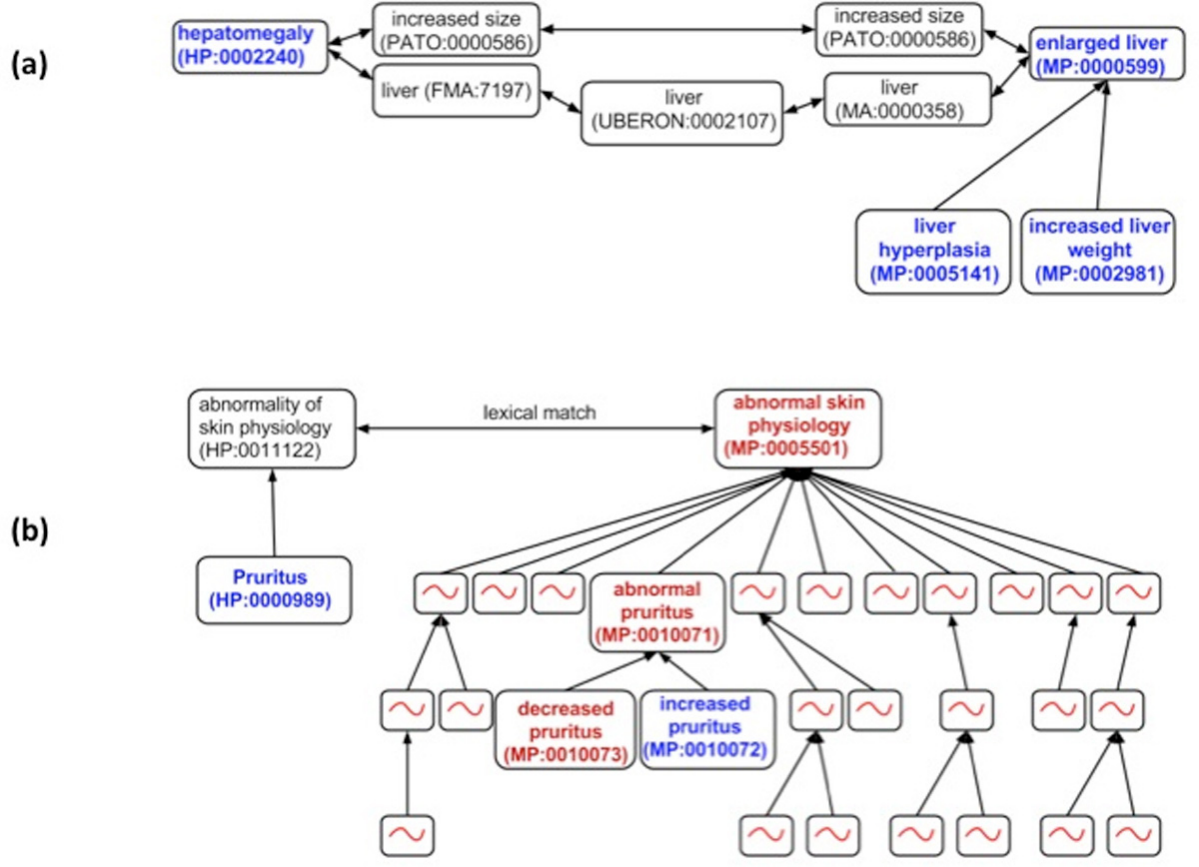
**Relationships between common HPO clinical phenotypes for the hepatic disease class and MP terms**. (a) The HPO term for *Hepatomegaly *is identified as being equivalent to the MP term for *enlarged liver *via their logical definitions. This MP term and its children are identified as the best scoring matches. (b) Lack of a logical definition for *Pruritis *leads to the best scoring MP matches being to the higher level term of *abnormal skin physiology *and all of its multiple child terms, many of which having no relationship to pruritis.

In contast, to some of the other disease categories, the ten most frequently occurring phenotypes were all consistent for hepatic disease rather than some being a consequence of Orphanet's classification of certain diseases to multiple categories. Eight of these most frequent occurring clinical phenotypes recalled the expected mouse phenotype as the best match in MP (and their child terms if they existed). For example, Figure 2a shows PhenoDigm results in *Hepatomegaly *matching *enlarged liver, liver hyperplasia *and *increased liver weight *as the best mouse phenotypes.

The other two concepts mapped sub-optimally or produced completely misleading results. For example, Figure 2b shows how the HPO concept *Pruritus *is matched to *abnormal skin physiology *as the best hit in mouse as well as all its child terms due to the lack of logical definitions for *Pruritus *(HP:0000989) and *increased pruritus *(MP:0010072). Although the 28 child terms include the ideal match, the additional matches will lead to non-specific mouse models being recovered. Finally, the best matches for the HPO concept *Elevated hepatic transaminases *(HP:0002910) were *increased liver copper level *and *increased liver iron level *based purely on increased concentrations of any object in the liver. Even though a corresponding MP concept exists, *increased circulating aspartate transaminase level *(MP:0005343), the correct logical definitions do not yet exist for PhenoDigm to have identified this relationship.

Although the performance could be improved if accurate logical definitions were created for the two poorly-mapped phenotypes, the fact that the others matched the expected mouse phenotypes as the best hit suggests that the poor overall performance of PhenoDigm in this disease category may be due to mouse mutants of hepatic disease genes not recapitulating the same phenotypes.

#### Neurological diseases

In previous studies it has been found that there are sufficient commonalities between humans and mice to determine disease gene candidates for some of the diseases belonging to the category of neurological diseases, e.g. diseases related to addiction [21]. However, there are still differences between mice and humans related to gene structure and spatiotemporal expression patterns that may prevent mice being in general applicable to neurological diseases [12]. Despite a mouse model not faithfully recapitulating a human disease, the mouse model may still provide insights into the origin of the disease [11].

The ten most commonly occurring HPO annotations in this category included two that do not have a neurological basis but are due to Orphanet's co-classification of diseases: *Short stature *and *Scoliosis*. However, both match the expected terms as the best hit in MP and their inclusion would therefore not be expected to account for the relatively poor performance of PhenoDigm for this disease category.

Looking at just the eight neurological phenotypes, four match the expected terms in MP and their child terms where present: *Seizures, Muscular hypotonia, Microcephaly *and *Nystagmus*. The other four only match high level terms in MP and all their child terms as the best scoring hits: *Cognitive impairment, Intellectual disability, Global developmental delay *and *Hyperreflexia*. These multiple matches lead to an imprecision when mouse models are ranked according to their phenotype similarity with the disease. This potentially leads to noisy results as multiple models are associated that are not necessarily relevant for the disease but due to the misaligned phenotypes. These issues in semantically mapping behavioural phenotypes may account for a large proportion of the poor performance in this disease category as opposed to underlying problems with using mice to model the biology of neurological diseases.

### Investigation of zebrafish model prediction results

To identify reasons for the differences in performances with respect to the applied disease categories, we further investigated the following three categories of diseases: *cardiac malformations, immunological *and *bone *diseases. We studied their annotations and the corresponding matches from PhenoDigm and the results are summarised in Additional File 1.

#### Cardiac malformations

The main reason for zebrafish's adoption as a model organism is the translucency of the organism in the embryonic stage, allowing *in vivo*, non-intrusive visualisation of organs as well as biological processes. Therefore, zebrafish are ideal model systems for studying developmental diseases and this may go some way to explaining why zebrafish outperformed mouse as a model of the congenital cardiac malformations in our analysis.

We observe that the most common clinical phenotype annotations in this disease category are matched efficiently by PhenoDigm. For example, *Abnormality of the aorta *(HP:0001679) matches various more specific aortic abnormalities in the Zebrafish Phenotype Ontology (ZP) annotations, whilst the clinical phenotypes *Defect in the atrial septum *(HP:0001631) and *Ventricular septal defect *(HP:0001629) are best aligned with the zebrafish phenotype *abnormally closed atrioventricular node. Tetralogy of Fallot *(HP:0001636) in itself comprises four separate phenotypes and here only matches *abnormally aplastic ventricular endocardium epithelium *as the best hit which is only a close association at best. In contrast, *Patent ductus arteriosus *(HP:0001643) does not match any sensible zebrafish phenotype but closure of the ductus arteriosis on birth, allowing the lungs to get their own supply of blood, is known to be a specific aspect of air-breathing vertebrates [22].

Many of the non-cardiac associated annotations seen in this disease category match less well to the fish phenotypes but are also less commonly seen and the cardiac matches alone appear to have been enough to efficiently recall the correct zebrafish models for most cardiac malformations. For example, no match to *Cognitive impairment *(HP:0100543) is retrieved and *Microcephaly *(HP:0000252) is aligned with *abnormally decreased thickness cranial nerve VIII *as the best match. Future investigation of why zebrafish phenotypes such as *abnormally hypoplastic head *were not the best hit for *Microcephaly *and some of the learning/memory fish phenotypes were not picked up for *Cognitive impairment *may further improve recall.

#### Immunological diseases

Although the zebrafish immune system closely approximates that of mammals, the main use of zebrafish in immunology comes from the fact that the embryonic stage already has a fully competent innate immune system allowing contrasting studies with the adaptive system [23]. One explanation for the poor performance in this disease category is that human adult immune phenotypes from a mixture of innate and adaptive responses are being compared to zebrafish embryonic innate phenotypes. The most common clinical phenotypes seen in this category are *Splenomegaly *(HP:0001744) and *Hepatomegaly *(HP:0002240) and these match the expected ZP terms of *abnormally increased size spleen *and *abnormally increased size liver *as the best scoring hits. However, other common clinical annotations such as *Recurrent bacterial or respiratory infections *(HP:0002718, HP:0002205) are not matched to anything in the zebrafish annotations beyond generalized immune system abnormalities. Recurrent infections suggest a long-lasting loss of protective immunity due to a perturbance in the adaptive immune system and as described above, this would not be observed in the embryonic zebrafish stages.

Other common immunological annotations in this category include *Lymphadenopathy *(HP:0002716), *Anemia *(HP:0001903), *Neutropenia *(HP:0001875) and *Thrombocytopenia *(HP:0001873) but none of these match the expected phenotypes in the zebrafish as the best scoring hit using our approach. The fact that zebrafish lack lymph nodes explains the first one but there are fish annotated with *abnormally present in fewer numbers in organism nucleate erythrocyte , abnormally present in fewer numbers in organism neutrophil *and *abnormally present in fewer numbers in organism thrombocyte*, so it would be expected that the clinical phenotypes should have recalled these fish phenotypes. Investigation and restructuring of the underlying ontologies and/or logical definitions to pick up these matches would presumably lead to an improvement in performance for this disease category.

#### Bone diseases

Despite having a different skeletal organisation, zebrafish has recently emerged as a useful complementary model for bone research due to the ability to study *in vivo*, processes such as osteogenesis and mineralization thanks to the existence of osteoblast-specific reporter lines [24].

Using PhenoDigm, the performance was mid-range for this disease category relative to the others. Looking at the most common skeletal clinical phenotypes we find that some match to the equivalent concepts in zebrafish whilst others warrant further attention. For example the most common clinical phenotype, *Short stature *(HP:0004322), is completely mis-matched to *abnormally decreased height enterocyte *as the best match. Fixing our approach such that *abnormally decreased length whole organism *is the best match would probably lead to a dramatic increase in performance. Other matches such as *Scoliosis *(HP:0002650) with *abnormally curved lateral vertebral column, Micrognathia *(HP:0000347) with *abnormally aplastic dentary *and *Brachydactyly syndrome *(HP:0001156) with *abnormally aplastic pectoral fin skeleton *are reasonable considering the evolutionary distance.

## Conclusions

Exomiser is a tool to narrow down gene candidate lists that have been identified in exome analyses using cross-species phenotype comparisons amongst other sources of evidence. Here we investigated the underlying PhenoDigm algorithm for different disease categories to understand where the approach is currently working well and to identify areas for further improvement. We demonstrated that the phenotype comparisons work better for some disease categories than for others. Furthermore, the prediction results depend on the organism and when automatically predicting disease gene candidates careful consideration is required as to which organism to apply for the predictions. However, it is somewhat difficult to disentangle whether performance differences exist due to differences in biology, the annotation methods used for each species or the focus of annotations for mouse and fish.

In addition to the identified biological restrictions that partially mirror community perceptions of how well the model organism can be fitted to human diseases, we showed that the underlying methodology still needs improvements. Even though a lot of work has been done in this direction, more logical definitions are needed in addition to improving the quality of the existing definitions to improve semantic mapping between the species-specific phenotype ontologies. Future work, will focus on improving these definitions and will undoubtably lead to improvements in the performance of PhenoDigm and Exomiser.

Even with a perfectly aligned set of phenotype ontologies, our results highlight that it will be dangerous to discount a model just because it does not perfectly match all the clinical phenotypes of the disease. For example, matches to clinical phenotypes such as lymphadenopathy were not seen in our assessment of the zebrafish results due to the lack of lymph nodes in fish rather than our alignment approach. In addition, different areas of interest of the researchers who phenotype the models need to be taken into account when using model organism to understand the genetic basis of disease i.e. particular phenotypes may not have been assessed. In conclusion, smarter tools are required that take into account the differences between species and accumulate predictions not only over multiple species but apply a sorting with respect to the applicability of the species in the particular area of disease.

## Methods

### Benchmark data: MorbidMap and Orphanet

Assessing the performance of a gene prediction or prioritisation algorithm requires benchmark data containing established gene-disease associations. One database containing manually confirmed associations between human diseases and genes is OMIM [1]. The human-centric gene-disease associations from OMIM are available via a download file called MorbidMap [25]. In addition we used the disease-gene associations curated by Orphanet. Both OMIM and Orphanet have HPO annotations that can be used by PhenoDigm and both were downloaded on 20 July 2013 and Mouse Genome Database (MGD)'s orthology file (see [26]) was used to convert the genes into mouse-specific gene identifiers that can be used for evaluation purposes. The final dataset contained a total of 3,429 diseases associated with 2,662 unique genes, which mapped to 2,772 orthologous genes in mouse and 1862 in fish.

### Generating prediction results with PhenoDigm

PhenoDigm [4] uses phenotype descriptions of human heritable disease and individual animal models to predict potentially gene candidates that may be causative for a diseases. The PhenoDigm algorithm uses a pairwise semantic similarity based on phenotype ontology annotations, such as HPO or MP, and prioritises genes according to this similarity measure. Applying the PhenoDigm method, a database was generated containing all the results displayed in the online web interface [27]. Instead of regenerating the data, we used the data built from 20 July 2013 so that the results presented here correspond with the current publicly available data.

### Dividing diseases into sets according to Orphanet categorisation

To divide the disease into sets that are biologically meaningful, we downloaded the Orphanet categorisation files from the Orphanet data download page [19] on 18 July 2013. We downloaded and processed 31 data files, one for each of the high level disease categories in the Orphanet categorisation. Each of the files contains a number of diseases that may or may not be referenced to OMIM. Furthermore, a disease may not only be assigned to one category and instead be mentioned in multiple files. For example, X-linked myotubular myopathy (OMIM:#310400) is categorised as a rare eye and neurological disorder because the most prominent symptoms include weakness, hypotonia and respiratory failure, as well as external ophthalmoplegia.

We note here that the Orphanet web interface also provides a category of *sucking/swallowing disorders*. This disease category was not included here as no categorisation file was provided on the Orphanet download page [19].

### Assessing PhenoDigm's performance according to disease categories

To determine PhenoDigm's performance, we applied ROC curves based on the gene-disease associations (see *Benchmark data: MorbidMap and Orphanet *). We divided the diseases into sets according to Orphanet's categorisation (see *Dividing diseases into sets according to Orphanet categorisation*) and consequently generated 31 evaluation sets. PhenoDigm's ranking was then compared using the 31 evaluation sets corresponding to each of the disease categories by determining true and false positive counts individually for each rank. As true positive counts a gene that is associated to a disease in MorbidMap or Orphanet. Conversely, a gene that is not mentioned in MorbidMap or Orphanet for a particular disease counts as false positive. We note here, that gene-disease associations may be counted as falsely identified connections, even though there is a relationship but it is not yet confirmed. However, we assume that this number is relatively small compared to the large number of possible combinations of genes and diseases and assume that our evaluation procedure is still appropriate. As a consequence, the true predictive rates provided here may be lower than they are in reality. To test the significance of each ROC analysis we performed 50 simulations per disease category where a set of diseases of the same size as the evaluation set was randomly chosen. These simulations provided a mean and standard deviation for the random distribution of scores for each evaluation set and these were used to calculate a p-value for the obtained result.

### Manual assessment of six disease categories

Further investigations into individual disease categories were necessary to identify potential shortcomings in either method or data. We chose six categories of diseases based on the worst, best, and one intermediate AUC score for each species (mouse and zebrafish). Two curators assessed the phenotype matches to either mouse or fish (see Additional File 1) for the ten most frequently occuring phenotypes accumulated over all the diseases falling into this category. The matches were assessed with respect to their biological correctness and whether they were sufficiently suitable to identify models from the respective organism.

## List of abbreviations

Mouse Genetics Project (MGP), Mouse Genome Database (MGD), Online Mendelian Inheritance in Man (OMIM), Mammalian Phenotype Ontology (MP), Human Phenotype Ontology (HPO), Receiver Operating Characteristic (ROC), Area Under Curve (AUC), Standard Operating Procedure (SOP),

## Competing interests

The authors declare that they have no competing interests.

## Authors' contributions

AOE and DS designed the study, as well as implemented all required scripts. CM developed the PhenoDigm software and NW, MH contributed annotation datasets. PR and SK contributed to the analysis of the results. All authors contributed to the final manuscript.

## Supplementary Material

Additional file 1 **Annotations and their matches for urogenetial, hepatic and neurological diseases with respect to mouse and fish models**. Excel sheet that contains the HPO annotations for the six further investigated disease categories: urogenital, neurological, hepatic, cardiac malformations, bone and immunological. In addition to the ten most frequent HPO annotations, we included the best scoring semantic matches to the respective model organism (either MP or ZP) as well as the frequency of this annotation. Click here for file

## Declarations

Publication in this supplement was support by National Institutes of Health (NIH) grant [1 U54 HG006370-01]. This article has been published as part of the Journal of Biomedical Semantics, Volume 5 Supplement 1, 2013: Proceedings of the Bio-Ontologies Special Interest Group Meeting 2013. The full contents of the supplement are available online at http://www.jbiomedsem.com/supplements/5/S1.

This article has been published as part of *Journal of Biomedical Semantics *Volume 5 Supplement 1, 2014: Proceedings of the Bio-Ontologies Special Interest Group 2013. The full contents of the supplement are available online at http://www.jbiomedsem.com/supplements/5/S1.
